# An encephalitis-like syndrome in an adult with methylmalonic acidemia and hyperhomocysteinemia: A case report

**DOI:** 10.1097/MD.0000000000051467

**Published:** 2026-11-06

**Authors:** Rongjin Zhang, Xiaona Sheng

**Affiliations:** a Department of Psychosomatic Medicine, The Second Hospital of Hebei Medical University, Shijiazhuang, Hebei, China.

**Keywords:** autoimmune encephalitis, case report, GAD65 antibody, hyperhomocysteinemia, metabolic encephalopathy, methylmalonic acidemia

## Abstract

**Rationale:**

Methylmalonic acidemia with hyperhomocysteinemia may remain unrecognized until adulthood. When fever, altered consciousness, psychiatric symptoms, cerebrospinal fluid (CSF) abnormalities, and electroencephalographic slowing dominate the presentation, infectious or autoimmune encephalitis is often considered first. Glutamic acid decarboxylase 65 antibody positivity may further complicate the diagnostic interpretation.

**Patient concerns:**

A 56-year-old man presented with fever and slowed responses for 15 days, followed by disorganized speech, visual hallucinations, episodes of impaired consciousness, and pyramidal signs.

**Diagnoses:**

Total homocysteine was > 100 µmol/L, propionylcarnitine (C3) was 22.42 µmol/L, and the C3/acetylcarnitine ratio was 0.83. Urinary organic acid analysis indicated increased methylmalonic acid and methylcitrate. Serum and CSF glutamic acid decarboxylase 65 antibodies were positive (titers, 1:100 and 1:3.2, respectively); however, the remaining autoimmune encephalitis antibody panel was negative, magnetic resonance imaging was nonspecific, and whole-exome sequencing revealed no clearly pathogenic variant. The findings supported methylmalonic acidemia with hyperhomocysteinemia on biochemical grounds and were clinically suggestive of a cobalamin metabolism-related disorder.

**Interventions:**

He received vitamin B12, vitamins B1 and B6, folic acid, levocarnitine, intravenous immunoglobulin, empirical anti-infective treatment, mechanical ventilation, and intensive supportive care.

**Outcomes:**

Following concurrent metabolic, immunologic, anti-infective, and intensive supportive treatments, his clinical symptoms improved. Total homocysteine decreased to 16.04 µmol/L, C3 to 19.50 µmol/L, and the C3/acetylcarnitine ratio to 0.40; CSF protein decreased from 1.78 to 0.80 g/L. Because these interventions were delivered simultaneously, the clinical and biochemical changes could not be attributed to any single treatment. At 6-month telephone follow-up, the family reported no recurrent fever, hallucinations, disorganized behavior, or marked disturbance of consciousness.

**Lessons:**

In adults with an encephalitis-like syndrome, recurrent neurologic symptoms, anemia, pyramidal signs, and marked hyperhomocysteinemia should prompt early metabolic screening. An antibody result should be interpreted alongside the clinical phenotype, biochemical profile, imaging findings, and CSF data, rather than used alone to assign etiology.

## 1. Introduction

Methylmalonic acidemia (MMA) comprises a heterogeneous group of organic acidemias. In a patient with MMA and marked hyperhomocysteinemia, disorders of intracellular cobalamin metabolism and remethylation warrant particular consideration.^[1,2]^ Although these disorders are usually recognized in childhood, later-onset disease can first be diagnosed in adults and may involve the nervous system, mental state, hematologic system, kidneys, vasculature, or multiple organ systems.^[3–7]^

Fever, altered consciousness, hallucinations, cerebrospinal fluid (CSF) protein elevation, and electroencephalography (EEG) slowing appropriately prompt investigation for infectious and autoimmune encephalitis. A recent medicine report of combined MMA with hyperhomocysteinemia and fluctuating cognitive impairment illustrates the diagnostic complexity of adult disease.^[8]^ The difficulty arises when marked metabolic abnormalities are treated as incidental, or when an antibody result is accepted as the etiology before the full clinical syndrome has been accounted for.^[9,10]^ We describe an adult with an encephalitis-like syndrome and serum/CSF glutamic acid decarboxylase 65 (GAD65) antibody positivity in whom the longitudinal history and biochemical profile shifted the diagnostic emphasis toward a potentially treatable metabolic encephalopathy.

## 2. Case presentation

A 56-year-old man was admitted because of fever and slowed responses for 15 days, followed by disorganized speech and visual hallucinations for 2 days. His illness began with reduced speech, intermittent limb tremor, and low-grade fever. Cranial computed tomography at a local hospital was unremarkable, whereas chest computed tomography showed a small localized inflammatory lesion in the posterior segment of the right upper lobe. Fever and tremor improved transiently after empirical anti-infective treatment. Before admission, another episode of high fever was followed by disorganized speech and visual hallucinations centered on weddings, funerals, graves, and people coming to take him away.

The past history suggested that the current illness was not an isolated neuropsychiatric event. Ten years earlier, he had been treated elsewhere for limb tremor, weakness, inability to walk, and impaired consciousness, with subsequent improvement. Two years earlier, he had been hospitalized for lower-limb weakness, dizziness, weight loss, pancytopenia, and mixed anemia. Bone marrow examination reportedly showed mixed anemia with impaired megakaryocyte maturation; he thereafter received folic acid and vitamin B12. There was no known family history of hereditary disease.

At admission, his temperature was 39.0°C. He had episodes of impaired consciousness and confused speech. Muscle-strength testing was limited by poor cooperation. Muscle tone was increased in all limbs, tendon reflexes were brisk, and bilateral Babinski signs, Hoffmann signs, palmomental reflexes, and neck stiffness were present. After correction of admission-related dehydration, liver and renal function were clinically unremarkable. High-sensitivity C-reactive protein was 72.6 mg/L, whereas procalcitonin and the complete blood count did not suggest severe bacterial infection. Blood cultures, fungal tests, viral screening, a respiratory pathogen antibody panel, bacterial toxin assay, tuberculin test, CSF culture, and CSF metagenomic testing did not identify a definite pathogen.

Initial lumbar puncture showed an opening pressure of 200 mmH_2_O, CSF protein of 1.78 g/L, and a white cell count of 1 × 10^6^/L. On repeat testing, the opening pressure was 130 mmH_2_O and CSF protein was 0.80 g/L; the white cell count remained 1 × 10^6^/L. Video EEG revealed a moderate-to-severe diffuse abnormality, with background slowing and left temporal delta activity. Brain magnetic resonance imaging (MRI) showed mild nonspecific white matter change in the right corona radiata without diffusion restriction (Fig. 1).

**Figure 1.**
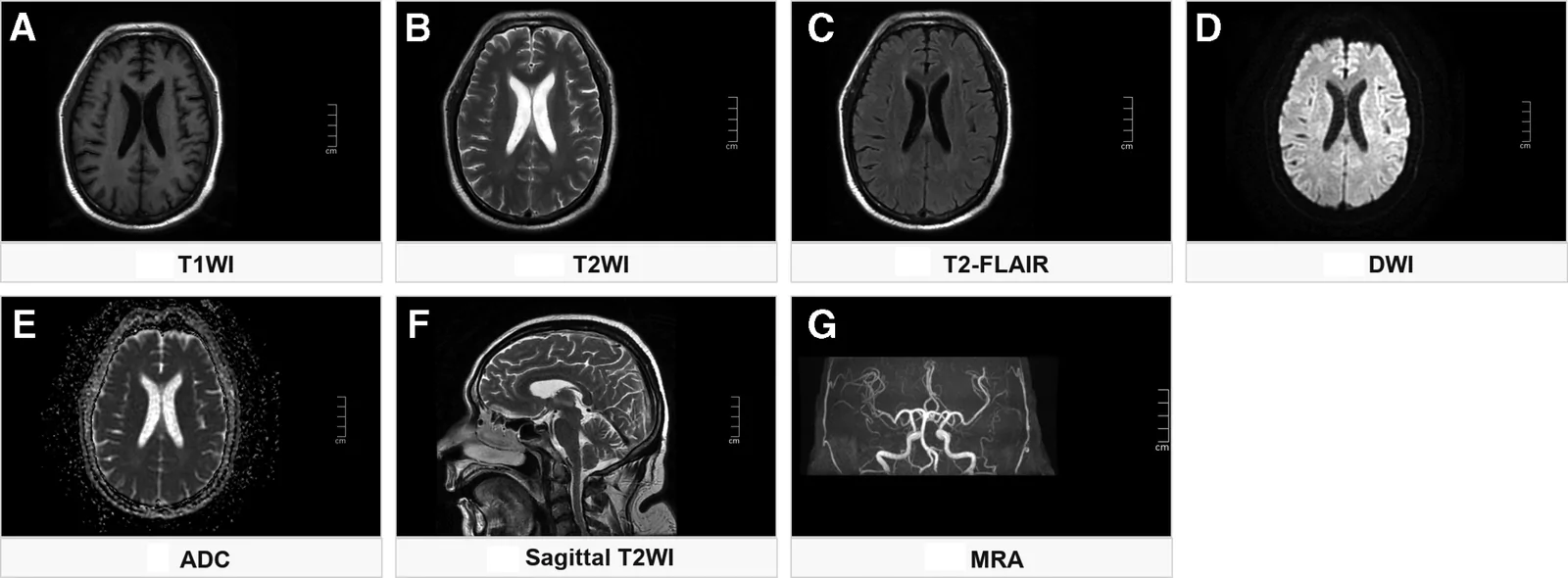
Brain magnetic resonance imaging findings. Mild nonspecific white matter changes were observed in the right corona radiata; diffusion-weighted imaging showed no definite diffusion restriction. (A) T1-weighted imaging; (B) T2-weighted imaging; (C) T2-fluid-attenuated inversion recovery; (D) diffusion-weighted imaging; (E) apparent diffusion coefficient map; (F) sagittal T2-weighted imaging; (G) magnetic resonance angiography. T1WI = T1-weighted imaging; T2WI = T2-weighted imaging; T2-FLAIR = T2-fluid-attenuated inversion recovery; DWI = diffusion-weighted imaging; ADC = apparent diffusion coefficient; MRA = magnetic resonance angiography.

Metabolic studies redirected the evaluation. Total homocysteine was > 100 µmol/L and 74.9 µmol/L on repeat testing; blood ammonia was 44 µmol/L. Dried blood spot acylcarnitine analysis showed a C3 concentration of 22.42 µmol/L (reference range, 0.30–5.00), a C3/acetylcarnitine ratio of 0.83 (reference range, 0.02–0.20), and a C3/methionine ratio of 0.85 (reference range, 0.02–0.30). Urinary organic acid analysis indicated increased methylmalonic acid and methylcitrate; methylcitrate-4(1) was 2.4 (reference range, 0.0–0.7) and methylcitrate-4(2) was 2.4 (reference range, 0.0–0.8).

Because of the psychiatric symptoms, altered consciousness, CSF protein elevation, and EEG slowing, autoimmune encephalitis was assessed in serum and CSF by tissue-based and cell-based indirect immunofluorescence assays. N-methyl-D-aspartate receptor, alpha-amino-3-hydroxy-5-methyl-4-isoxazolepropionic acid receptor 1/2, leucine-rich glioma-inactivated protein 1, contactin-associated protein-like 2, gamma-aminobutyric acid type B receptor, dipeptidyl-peptidase-like protein 6, immunoglobulin-like cell adhesion molecule 5, metabotropic glutamate receptor 5, and myelin oligodendrocyte glycoprotein antibodies were negative. Serum GAD65 immunoglobulin G was positive at 1:100, and CSF GAD65 immunoglobulin G was positive at 1:3.2. A paraneoplastic neurologic syndrome immunoblot was negative except for GAD65 positivity. Singleton whole-exome sequencing did not identify a phenotype-relevant sequence variant, copy-number variant, or mitochondrial circular variant classified as clearly pathogenic.

## 3. Diagnostic assessment

The main alternatives were infection, autoimmune encephalitis, and metabolic encephalopathy (Table 1). Fever, elevated hypersensitive C-reactive protein, and localized pulmonary inflammation supported the possibility of an infectious or inflammatory trigger. However, the negative pathogen work-up, normal CSF white cell count, and absence of a focal central nervous system infectious pattern made infection alone an insufficient explanation for the full neuropsychiatric and pyramidal syndrome. Infection was therefore considered a possible precipitating or coexisting factor rather than the unifying diagnosis.

**Table 1:** Diagnostic evidence supporting and limiting each etiological framework.

Diagnostic framework	Evidence supporting consideration	Evidence limiting a primary diagnosis	Interpretation in this case
Infectious encephalitis or infection-associated encephalopathy	Fever, elevated high-sensitivity C-reactive protein, and mild localized pulmonary inflammation on chest CT.	No definite pathogen; CSF white cell count 1 × 10^6^/L; metabolic abnormalities and pyramidal signs not explained by localized pulmonary inflammation alone.	Possible precipitating or coexisting factor, but insufficient as a unifying diagnosis.
Autoimmune encephalitis, including GAD65-associated disease	Psychiatric symptoms, altered consciousness, EEG slowing, CSF protein elevation, and serum/CSF GAD65 positivity.	No paired quantitative CSF/serum GAD65 assessment or documented intrathecal synthesis; other neuronal antibodies negative; no typical limbic MRI pattern.	A relevant immune differential diagnosis and reason for empirical IVIG, but not sufficient to establish anti-GAD65 autoimmune encephalitis.
Metabolic encephalopathy related to MMA with hyperhomocysteinemia	Prior recurrent neurologic episodes and mixed anemia; homocysteine > 100 µmol/L; elevated C3 and C3/C2; increased methylmalonic acid/methylcitrate by urinary organic acid analysis; treatment-associated biochemical improvement.	No clearly pathogenic variant on whole-exome sequencing; directly reportable quantitative blood/urinary methylmalonic acid values unavailable.	Biochemically supported MMA with hyperhomocysteinemia, clinically suggestive of a cobalamin metabolism-related disorder.

C3 = propionylcarnitine, C3/C2 = propionylcarnitine/acetylcarnitine ratio, CSF = cerebrospinal fluid, EEG = electroencephalography, GAD65 = glutamic acid decarboxylase 65, IVIG = intravenous immunoglobulin, MMA = methylmalonic acidemia.

Autoimmune encephalitis remained a reasonable consideration because of the acute neuropsychiatric syndrome, CSF protein elevation, and EEG slowing. Detection of GAD65 immunoglobulin G in serum and CSF justified immune evaluation and empirical treatment during diagnostic uncertainty but did not establish anti-GAD65 autoimmune encephalitis. Current diagnostic frameworks require antibody findings to be interpreted in relation to the clinical syndrome, MRI and CSF evidence, and exclusion of alternative causes.^[9,10]^ In this patient, the remaining neuronal antibody panel was negative, MRI did not show a characteristic limbic pattern, and the metabolic profile provided a more persuasive alternative explanation. GAD65 positivity was consequently treated as a competing diagnostic clue, not the principal etiological diagnosis.^[11,12]^ However, paired quantitative serum-CSF GAD65 measurements and evidence of intrathecal antibody synthesis were unavailable. Therefore, GAD65-mediated autoimmunity could not be definitively excluded; the available findings only indicated that the antibody result was insufficient to establish it as the principal diagnosis.

Taken together, the recurrent neurologic symptoms and mixed anemia, marked hyperhomocysteinemia, elevated C3 and C3/acetylcarnitine ratio, and urinary increases in methylmalonic acid and methylcitrate provided a consistent longitudinal and biochemical pattern. The diagnosis was therefore reported as MMA with hyperhomocysteinemia, supported by biochemical evidence and clinically suggestive of a cobalamin metabolism-related disorder. The exome result prevented molecular subtyping but did not conflict with the biochemical diagnosis or the decision to initiate treatment. Figure 2 summarizes the diagnostic pathway.

**Figure 2.**
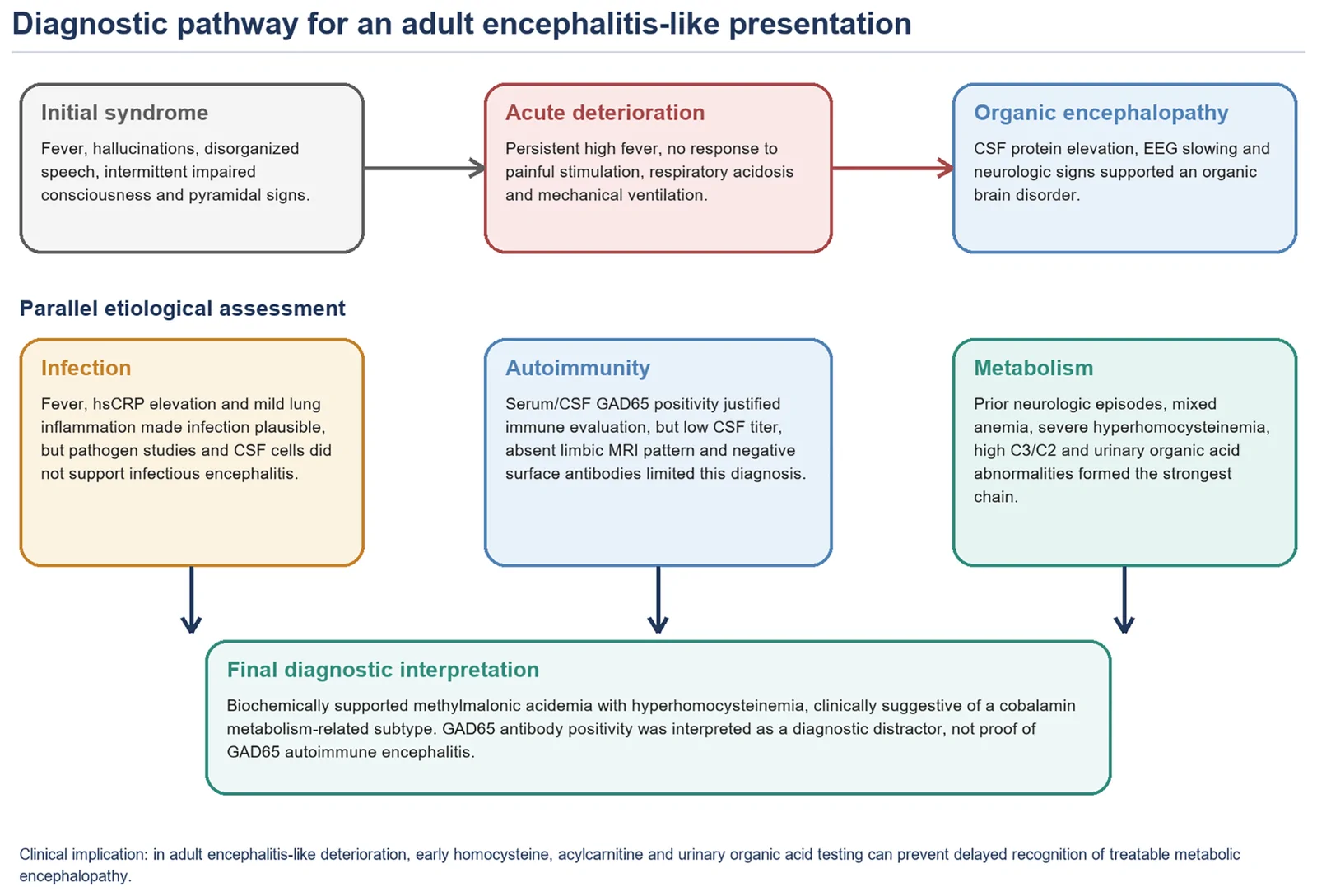
Diagnostic pathway in the present case. Infection and autoimmunity were reasonable considerations at presentation. Integration of prior neurologic and hematologic history with severe hyperhomocysteinemia, acylcarnitine abnormalities, urinary organic acid abnormalities, CSF/EEG findings, and treatment-associated biochemical improvement supported a metabolic encephalopathy pathway. CSF = cerebrospinal fluid, C3/C2 = propionylcarnitine/acetylcarnitine ratio, EEG = electroencephalography, GAD65 = glutamic acid decarboxylase 65, hsCRP = high-sensitivity C-reactive protein, MRI = magnetic resonance imaging.

## 4. Treatment and outcome

During the parallel infectious, immune, neurophysiologic, imaging, genetic, and metabolic work-up, the patient remained febrile and became unresponsive to painful stimulation. Arterial blood gas analysis showed hypercapnic respiratory acidosis (pH 7.28, pCO_2_ 59 mm Hg, pO_2_ 89.9 mm Hg, base excess 0.8, and lactate 1.1 mmol/L). He underwent tracheal intubation and was transferred to the intensive care unit. With mechanical ventilation, temperature control, nutritional support, and fluid-electrolyte management, his consciousness gradually improved, and he was extubated.

Treatment for the suspected metabolic disorder consisted of vitamin B12 1 mg intramuscularly once daily, vitamin B1 100 mg intramuscularly once daily, vitamin B6 10 mg twice daily, folic acid 5 mg 3 times daily, and oral levocarnitine solution 1 g 3 times daily. Because autoimmune encephalitis remained in the differential diagnosis, intravenous immunoglobulin (IVIG) was administered at 30 g/d for 4 consecutive days. Empirical anti-infective, antiviral, and symptomatic treatments were given concurrently during the acute phase.

Following treatment, slowed responses, disorganized speech, and hallucinations improved. Total homocysteine decreased to 16.04 µmol/L, C3 to 19.50 µmol/L, and the C3/acetylcarnitine ratio to 0.40. CSF opening pressure and protein concentration also decreased. Because B-vitamin-based metabolic therapy, levocarnitine, IVIG, anti-infective agents, mechanical ventilation, and intensive supportive care were administered concurrently, neither the clinical nor the biochemical improvement could be attributed to any single intervention. Serial clinical, biochemical, and CSF findings are summarized in Table 2. At telephone follow-up at 2 weeks, 1 month, and 6 months, the family reported that he continued levocarnitine and B vitamins; there had been no recurrent fever, hallucinations, disorganized behavior, or marked disturbance of consciousness, and his gait had improved. Repeat urinary organic acid analysis, EEG, brain MRI, CSF antibody testing, and long-term metabolic monitoring were not available.

**Table 2:** Key biochemical, cerebrospinal fluid, and clinical changes during treatment and follow-up.

Variable	Before or early during admission	After treatment or follow-up	Clinical interpretation
Total homocysteine	>100 µmol/L; repeat 74.9 µmol/L	16.04 µmol/L	Marked decrease during combined treatment.
Propionylcarnitine (C3)	22.42 µmol/L (reference range, 0.30–5.00)	19.50 µmol/L	Decreased but remained above the reference range.
C3/C2 ratio	0.83 (reference range, 0.02–0.20)	0.40	Improved but remained abnormal.
Urinary organic acids	Methylmalonic acid and methylcitrate increased by report conclusion; methylcitrate-4(1), 2.4 (reference range, 0.0–0.7); methylcitrate-4(2), 2.4 (reference range, 0.0–0.8)	Not repeated	Supported MMA-related biochemical abnormality.
CSF pressure and protein	200 mmH_2_O; protein 1.78 g/L; white cell count 1 × 10^6^/L	130 mmH_2_O; protein 0.80 g/L; white cell count 1 × 10^6^/L	CSF protein and pressure decreased during clinical improvement.
Clinical status	Fever, intermittent impaired consciousness, hallucinations, disorganized speech, pyramidal signs, and respiratory acidosis requiring mechanical ventilation	No recurrent fever, hallucinations, disorganized behavior, or marked consciousness disturbance during telephone follow-up at 0.5, 1, and 6 months; gait improved	Clinical improvement occurred under combined metabolic, immune, and supportive treatment; single-treatment causality cannot be assigned.

C3 = propionylcarnitine, C3/C2 = propionylcarnitine/acetylcarnitine ratio, CSF = cerebrospinal fluid, MMA = methylmalonic acidemia.

## 5. Discussion

The diagnostic difficulty was not whether infection or autoimmunity merited assessment at presentation; both did. Rather, it was deciding when the accumulating evidence favored an underlying metabolic disorder. Fever, altered consciousness, hallucinations, CSF protein elevation, and EEG slowing appropriately prompted investigations for infectious and autoimmune encephalitis. The earlier neurologic episodes and mixed anemia, together with marked hyperhomocysteinemia and propionate-pathway abnormalities, placed the acute illness in a longer clinical context and were more readily reconciled by metabolic encephalopathy. Adult cobalamin-related disorders and MMA with hyperhomocysteinemia may present with psychiatric symptoms, cognitive impairment, gait disturbance, pyramidal signs, neuropathy or myelopathy, hematologic abnormalities, renal disease, pulmonary hypertension, and thrombotic complications.^[3–8]^

Neither the fever nor the respiratory acidosis can be attributed directly to MMA on the available evidence. The combination of persistent fever, worsening consciousness, and hypercapnic respiratory acidosis documented a severe encephalopathic episode requiring airway protection and intensive care. Infection, inflammation, or other physiological stress may have reduced the threshold for decompensation in a metabolically vulnerable patient. Catabolic stress, mitochondrial dysfunction, impaired mitophagy, cellular distress, and oxidative or inflammatory responses have been implicated in the neurological vulnerability associated with MMA.^[13,14]^ Addressing potential precipitants while starting metabolic treatment was therefore clinically appropriate.

GAD65 antibody positivity added a genuine diagnostic dilemma. Neurologic GAD65 autoimmunity is associated with stiff-person spectrum disorders, cerebellar ataxia, epilepsy, and limbic encephalitis, but the clinical meaning of a positive result depends on the antibody level, phenotype, compartment, assay context, and corroborating evidence.^[11,12]^ In this case, the result supported empirical immunotherapy while the diagnosis was unsettled, but it did not outweigh the metabolic data. An isolated antibody finding should not lead to prolonged immunotherapy when a potentially treatable metabolic cause has not been adequately addressed.^[10]^

Accordingly, the diagnosis should be reported at the biochemical rather than molecular level. Whole-exome sequencing did not identify a clearly pathogenic variant, so cblC disease or another molecular subtype cannot be described as genetically confirmed. The available evidence supports MMA with hyperhomocysteinemia on biochemical grounds and is clinically suggestive of a cobalamin metabolism-related disorder. Molecular definition remains important as disease-specific therapeutic approaches for organic acidemias continue to develop.^[15]^

Several limitations should be acknowledged. Objective laboratory data from the earlier admissions could not be reviewed. The organic acid report indicated increased methylmalonic acid, but direct quantitative blood or urinary methylmalonic acid values were unavailable; acute and historical vitamin B12 and folate concentrations were also unavailable. An acquired contribution to hyperhomocysteinemia therefore cannot be fully excluded, and the biochemical phenotype cannot be assigned to a specific cobalamin disorder. In addition, paired quantitative CSF/serum GAD65 measurements and other markers of intrathecal immune synthesis were not available, which limits interpretation of the antibody result. B-vitamin-based metabolic therapy, levocarnitine, IVIG, anti-infective agents, mechanical ventilation, and intensive supportive care were administered concurrently; therefore, neither the clinical course nor the biochemical changes permit attribution of efficacy to any single intervention. Finally, follow-up was based exclusively on telephone interviews with the family and was not supported by repeat homocysteine measurement, acylcarnitine profiling, urinary organic acid analysis, MRI, EEG, or antibody testing. Accordingly, the reported follow-up describes family-reported clinical stability rather than objectively verified long-term remission. These constraints limit causal and molecular inference, but they do not detract from the practical value of early metabolic screening in adults with an encephalitis-like syndrome.

## 6. Conclusions

Adults with an encephalitis-like syndrome who have recurrent neurologic symptoms, anemia, pyramidal signs, or marked hyperhomocysteinemia warrant early metabolic screening alongside infectious and autoimmune evaluation. In this patient, the longitudinal history, severe hyperhomocysteinemia, elevated C3 and C3/acetylcarnitine ratio, urinary organic acid abnormalities, and treatment-associated biochemical improvement favored MMA with hyperhomocysteinemia as the best unifying diagnosis. GAD65 positivity was clinically relevant but was insufficient to replace an integrated diagnostic assessment. His condition improved after comprehensive treatment, although the independent contribution of each intervention could not be determined. This case highlights the need for prompt, targeted metabolic screening in adults with acute encephalopathy of unclear cause, particularly when marked hyperhomocysteinemia or other metabolic abnormalities are present, to avoid diagnostic anchoring on a single immunologic finding.

## Acknowledgments

The authors thank the patient and his family for their cooperation. OpenAI Codex (GPT-5) was used to assist with language editing, formatting, and drafting support. All clinical information, literature citations, interpretations, and final content were independently reviewed and verified by the authors, who take full responsibility for the manuscript.

## Author contributions

**Conceptualization:** Rongjin Zhang, Xiaona Sheng.

**Data curation:** Rongjin Zhang.

**Investigation:** Rongjin Zhang.

**Supervision:** Xiaona Sheng.

**Writing – original draft:** Rongjin Zhang.

**Writing – review & editing:** Rongjin Zhang, Xiaona Sheng.
